# Supplementary material for: Training Packages and Patient Management Tools for Healthcare Staff Working with Small, Nutritionally At-Risk Infants Aged under 6 Months: A Mixed-Methods Study
Source: Children (Basel). 2023 Sep 1;10(9):1496. doi: 10.3390/children10091496 (PMC10530104; doi:10.3390/children10091496)
Supplement: Supplementary file 1 [file children-10-01496-s001.zip › children-2562773-supplementary.pdf]

Table S1 - Identified Trainings addressing mothers and at-risk infants <6 months with extended summary of curriculum

| | WHO | | | | | | | | | | | | | |
|---|---|---|---|---|---|---|---|---|---|---|---|---|---|---|
| Curriculum | Nutrition Works/EN/ IASC/ USAID | UNICEF | UNICEF | UNICEF | UNICEF | UNICEF/LINKAGE/ IBFAN/ENN | UNICEF/ UNAIDS | | ENN | LSHTM | SC | ACF | Holt International | CARE, URC/CHS |
| | Harmonised Training Package (HTP) | Breastfeeding Counselling a Training Course | Community IYCF Counselling Pack | IYCF counselling: An integrated course | IMCI | Infant feeding in emergencies | HIV and Infant Feeding Counselling | Think Healthy | C-MAMI | Getting to know cerebral palsy* | IYCF-E Toolkit | Baby friendly Spaces | Feeding and Positioning Manual: Guidelines for Working with Babies and Children | IYCF Counselling |
| **Infant** | | | | | | | | | | | | | | |
| Triage | | | | | | | | | x | | | x | | |
| Feeding Assessment | x | x | x | x | x | x | | | x | x | x | x | | x |
| Anthropometric / Nutritional Assessment | | | | | x | | | | x | | | x | | |
| Assessing Illness | | | | | x | x | | | x | | | | | |
| Growth Monitoring | | x | x | x | | x | | | x | | x | x | | x |
| Identify Treatment | | | | | x | | | | x | | | | | |
| Kangaroo Care | | | x | | | x | | | | | x | x | | |
| **The Sick Child** | | x | | | x | | | | | | | | | x |
| Diarrhoea | | x | | | x | | | | | | | | | x |
| SAM | | | | | x | x | | | x | | | | | |
| **Breastfeeding** | x | x | x | x | x | x | x | | x | x | x | x | x | x |
| Why it is important | | x | | x | x | x | x | | | | x | x | x | x |
| Best Practice | | x | | x | | x | | | | | x | x | | x |
| How it works | | x | x | x | | x | x | | | | x | x | x | x |
| Difficulties | | x | x | x | x | x | | | x | x | x | x | | x |
| Risk of not breastfeeding | | x | x | | | x | x | | | | | | | |
| IYCF | x | x | x | x | | x | | | | | x | x | | x |
| Artificial feeding | x | x | | | | x | | | | | x | x | x | |
| BF Beliefs | | | x | | | | | | | | x | x | | x |

| | | | | | | | | | | | | | | |
|---|---|---|---|---|---|---|---|---|---|---|---|---|---|---|
| BF Support | | x | x | x | | x | x | | | | x | x | | x |
| BF Counselling | | x | x | x | x | | | | | | x | x | | x |
| Expressing Breastmilk | | | | x | | | | | | | x | x | | x |
| Behaviour Change | | | x | | | | | | | | x | x | | x |
| Relactation | | x | | | | x | | | x | | x | x | | |
| How to breastfeed while working | | | | | | | x | | | | | | | |
| **Mother** | | | | | | | | | | | | | | |
| Maternal health assessment | | | | | | | | x | x | | x | x | | x |
| Women’s nutrition, health and fertility | | | | | x | x | | x | | | x | x | | x |
| Counselling the mother | | | | | x | x | | x | | | x | x | | x |
| Preparing for the baby / pregnancy | | | | | | | | x | | | | | | |
| **HIV and Infant Feeding** | | x | x | x | | x | x | | x | | x | x | x | x |
| Risk of mother child transmission (HIV) | | x | x | x | | x | x | | | | x | x | | x |
| decision making on feeding practice | | | x | x | | x | x | | | | x | | | |
| **Disability** | | | | | | | | | | x | | | x | |
| Feeding difficulties | | | | | | | | | | x | | | x | x |
| **Counsellors** | | | | | | | | | | | | | | |
| Counselling Skills | | x | x | x | x | x | x | x | | x | x | x | | x |
| Communication Skills | | x | x | x | x | x | | x | | | x | x | | x |
| BCC | | | | x | | | | x | | | x | x | | x |
| **Other** | | | | | | | | | | | | | | |
| Management of Artificial | x | | | x | | x | | | | | x | x | | |

| | | | | | | | | | | | | | | |
|---|---|---|---|---|---|---|---|---|---|---|---|---|---|---|
| feeding/ donations | | | | | | | | | | | | | | |
| Emergency Preparedness | x | | | | | x | | | | | x | x | | |
| Policies and Guidelines for appropriate infant feeding | x | | | x | | | | | | | x | x | | x |
| Food Hygiene | | x | | | | x | | | | | x | x | | |

*Table S2 - Comparing logistics of identified Trainings addressing mothers and at-risk infants <6 months*

| | WHO | | | | | | | | | | | | | |
|---|---|---|---|---|---|---|---|---|---|---|---|---|---|---|
| Curriculum | Nutrition Works/EN/ IASC/ USAID | UNICEF | UNICEF | UNICEF | UNICEF | UNICEF/LINKAGE/ IBFAN/ENN | UNICEF/ UNAIDS | | ENN | LSHTM | SC | ACF | Holt International | CARE, URC/CHS |
| | Harmonised Training Package (HTP) | Breastfeeding Counselling a Training Course | Community IYCF Counselling Pack | IYCF counselling: An integrated course | IMCI | Infant feeding in emergencies | HIV and Infant Feeding Counselling | Think Healthy | C-MAMI | Getting to know cerebral palsy* | IYCF-E Toolkit | Baby friendly Spaces | Feeding + Positioning Manual: Guidelines for Working with Babies and Children | IYCF Counselling |
| **Year published** | Vol. 2, 2011 | 1993 | 2010 | 2006 | 1997 | Module 2, Vol. 1.1, 2001 | 2000 | 2015 | v.2 2018 | | V. 2.1 2010 | 2014 | | 2007 |
| **Who is the training aimed at?** | Trainers in the Nutrition in Emergencies sector | Trainers training Health workers | CHW other CW, primary health care Staff | Lay Counsellors, CHW, PMTCT Counsellors, Nurses, Clinicians at first referral level | Doctors, Nurses, Health workers | Health and Nutrition Workers | Guide to train Trainers, CHW working with HIV+ mothers and babies | CHW | Health Worker | facilitator, parents, caregivers and persons with cerebral palsy | Emergency relief Staff, technical Staff, Nutrition Advisors, Coordinators and programme managers | Psychologists, Psychosocial workers, Animators, Lactation Counsellors, Midwives, Nurses, Community health workers | Caregivers | low literacy, community-level Infant and Young Child Feeding (IYCF) Counsellors |
| **What target population is addressed?** | Infants and children affected by emergencies (no age specification) | Women and children after the perinatal period | infants 0-24 months | Mothers with Infants 0-24 months | infants 1 week up to 2 months / children 2 months - 5 years | Mothers / Caregivers of Infants | Mothers and babies (no age specification) in areas with high prevalence of HIV | Mothers of infants | at-risk Mothers and Infants under 6 months | Children that suffer from cerebral palsy | Mothers and infants from 0-60 months | Pregnant and Lactating Women and their Children | Infants 0-12 months | Mothers and Infants 0-23 months |
| **What setting is** | In emergencies | In the Community | In the community | / | Inpatient and | Emergency | In the community | In the community | In the community | In the community | In Emergencies | In Emergencies | / | Low Resource Settings |

| | | | | | | | | | | | | | | |
|---|---|---|---|---|---|---|---|---|---|---|---|---|---|---|
| **the training aimed at?** | | | | | Outpatient Care | | | | | | | | | |
| **How long is the training (how many hours)?** | / | 40 h | 20 sessions x 1.4h / 5 Days | 5 days | 11-day course | / | 16 Sessions 3-day course approx. 6 contact h each day (17h 45min) | min 5 days target duration 10 days | / | dependent on the setting, can be comprised into 2-3 days | Dependent on setting, no specification | Dependent on setting, no specification | / | 5 Days |
| **How is the training delivered?** | Resource package that can be used by trainers during process of course development. Not a ready to use training course itself. | Lectures, demonstrations, small group discussions, reading, role-play, exercise | From Facilitator | Classes and Practice | classes and clinical sessions | Classes, Case Studies | contact | from trainers to CHW | Tool for health workers | Participatory training | e-Training, Contact | Contact | Manual is intended to be used by caregivers as guide on feeding practices | Variety of Training Methods, Counselling, Material, Visual Aids, demonstrations, Group discussions, Case Studies, Role plays, Practice |
| **Are there any toolkits provided? / Accompanying course material?** | / | Participants manual Answer sheet Forms and Checklist for clinical practice and counselling, Story cards, Videotapes | IYCF counselling package, Facilitator Guide, Training Aid, Participant Handouts and, monitoring tools, IYCF counselling Cards, Key messag, | Directors and Trainers, Guide, Participants Manual, Follow-up Guidelines, Slides presentation, Answer Sheets, Forms Checklists, Story Cards | Director and Facilitator Guide, outpatient clinical practice Guide, inpatient, Comprises 15 self-learning Booklets | Manual, Overhead Figures, Presenters Notes, | Directors Guide, Trainers Guide, Overhead Transparencies, Participants manual Feeding options card | reference manual Health calendar | Counselling booklets counselling cards, C-MAMI programme cards | / | Guide on common BF myths, Assessment Tools, Guides, Implementation Tools, Counselling Cards, Course Materials, Videos | / | / | Counselling cards, Message Booklet, Trainers Guide, no dependence on slides or media projection |

| | | | | | | | | | | | | | | |
|---|---|---|---|---|---|---|---|---|---|---|---|---|---|---|
| | | | Booklet, take home Brochures, Planning - Adaptation guide | | | | | | | | | | | |
| **Does the training include an implementation guide?** | Summarizes topics that must be considered when implementing, such as staffing, | Directors Guide | Includes a planning Guide | Yes | | No | | | | | | Yes | | Includes Clear instructions for facilitator on training delivery |
| **Does the Training include a Referral Guide** | Mentions to establish referral to skilled IYCF assistance | Covers signs of when referral is needed | Covers signs of when referral is needed | Yes | | Yes | | | referral Guide based on symptoms, and WFA, WFL | | | Yes | | Yes |
| **Is an end evaluation included?** | Module on monitoring and evaluation | / | Post training follow ups by facilitator max. 3 months after training | Follow up Guidelines after Training | / | No | | / | / | short section about monitoring and evaluation of the training | / | Monitoring and Evaluation process. Tools for measuring outcome and impact indicators | / | evaluation of participants knowledge and satisfaction with training |
| **Certificate successful completion** | / | / | / | / | / | / | | / | / | / | / | / | / | / |
| **Available languages** | English | English, French, Russian | English | English, Spanish, Russian | English | English | English | English, French, Spanish, Turkish | English | English, French, Spanish | English, French, Arabic | English, French, Arabic | English | English |
